# 

**Published:** 1868-06

**Authors:** 


### IOWA STATE DENTAL SOCIETY.

The Iowa State Dental Society will hold its fifth annual session in the city of Keokuk, commencing on Tuesday evening, July 21, 1868, and continue two whole days. We cordially invite all our professional brethren to be present, and aid in making the occasion one of interest and mutual profit.

The following gentlemen have been appointed to prepare and read Essays on the occasion: Dr. H. S. Chase, St. Louis, "Best mode of preserving in health the Dental Pulp after exposure." Dr. H. D. Bronson, "Anæsthetics." Dr. J. Hardman, "Some reasons for the early extraction of the first or six year molar." Dr. W. O. Kulp, "Saliva." Dr. A. B. Mason, "Origin and formation of the Teeth, and what is best adapted to their healthy growth." Dr. P. T. Smith, "The Dental Tissues." Dr. M. L. Jackson, "Filling Teeth." Dr. I. P. Wilson, "Dental Quackery."

Prof. Hughes, of Keokuk Medical College, has been invited to deliver a Lecture during the session.

Any one having specimens of malformation, or cases of special interest to the profession, is requested to report the same. Do not let business prevent you from attending. Come expecting a good time, and contribute to the interest of the Association.

J. F. Sanborn, *President.*

N. H. Tulloss, *Corresponding Secretary.*

### AMERICAN DENTAL ASSOCIATION.

THE annual convocation of this body takes place on Tuesday, the 28th of July, at Niagara Falls. That is a central point, and one too where every one delights to go, at every opportunity and especially in the warm season. These things, together with the interest existing in the profession for this Association, will doubtless draw together a large number, and we feel well assured that there will be a very interesting time. It is the duty of every member to go up to these meetings, fully prepared to contribute what he may for the advancement of our profession; and in order to do this he should be very familiar, not only with the history of the professsion, but with its present status and tendencies, that the good may be encouraged and the bad opposed and counteracted.

We would not have every one, nor even any one go with some fixed notion in mind which he would thrust forward obtrusively, in season and out of season. But whatever any one may present that is really valuable, the majority will be interested to secure. It is genuine selfishness that prompts the remark, "Oh, I come to receive, not to give." We trust there will be a good meeting, and that all parts of our country may be represented. We would suggest to all who may have reports or papers prepared, that if they are unable to be present, they should send along the documents. T.

### FRIENDLY SUGGESTIONS.

We presume it is a common thing with those who perform a large amount of unrequited labor, to receive numerous suggestions from anxious friends as to how the work might be better done. "We know they are friends from the remarks they make."

Recently it was suggested to us that the REGISTER ought to be more dignified; by another, that we ought to introduce more short, spicy articles; by another, that it enters too much into practical details; by another, that principles are too much discussed in it; one suggests, that we publish *too* much proceedings of societies; another is quite exercised that the proceedings of his society do not appear as he would have them; one says the cover is not the right color; another says we don't mail it right; one says it ought to look more like this journal, another like that.

Now, we take all these suggestions in the kindest manner possible, and follow them all, just so far as we can, and have the DENTAL REGISTER left. We receive these suggestions as the wishes of our friends, that we shall be a great deal better than we are. We prefer to be just what we are, rather than to be somebody else. We love individuality, and we propose in the future to edit the DENTAL REGISTER, and not something else. Nevertheless, we shall be thankful for every hint or suggestion that will tend to make it a better DENTAL REGISTER. Now, come on and help us. T.

### The Dental Register

OF THE WEST.

*Issued Monthly, at $3 a Year in Advance.*

DEVOTED TO THE INTERESTS OF THE DENTAL PROFESSION.

EDITED BY J. TAFT AND G. WATT.

The volume commences in January and closes in December.

The Register being issued monthly is always fresh, therefore indispensable to the practitioner who desires to keep posted up with the new inventions and improvements which are daily developed. Neither expense nor effort will be spared to give value and variety to its contents. Articles will be illustrated with well executed engravings whenever they will add to the interest or assist in explanation.

Its extensive circulation and frequency of issue makes it one of the BEST DENTAL ADVERTISING MEDIUMS in the United States.

RATES OF ADVERTISING.

One page, 1 year, $50. Half page, 1 year, $30. Quarter page, or card, 1 year $20.

All advertising bills payable in advance, or at furthest after the issue of the first number containing the advertisement. All transient advertisements to be paid for in advance.

☞ CONTRIBUTIONS SOLICITED.

Address, J. TAFT, or "DENTAL REGISTER," Cincinnati Ohio.

Business Communications to

**J. TAFT, Publisher,**

No. 238 Fourth St., between Plum and Central Avenue

